# Transition of LINE-1 DNA Methylation Status and Altered Expression in First and Third Trimester Placentas

**DOI:** 10.1371/journal.pone.0096994

**Published:** 2014-05-12

**Authors:** Zhi-ming He, Jinping Li, Yi Lisa Hwa, Brian Brost, Qun Fang, Shi-Wen Jiang

**Affiliations:** 1 Fetal Medicine Center, Department of Obstetrics and Gynecology, The First Affiliated Hospital of Sun Yat-Sen University, Guangzhou, Guangdong, China; 2 Department of Biological Science, Mercer University School of Medicine, Savannah, Georgia, United States of America; 3 Department of Obstetrics and Gynecology, Mayo Clinic and Mayo College of Medicine, Rochester, Minnesota, United States of America; 4 Department of Obstetrics and Gynecology, Memorial Health University Medical Center, Savannah, Georgia, United States of America; 5 Department of Medicine, Mayo Clinic and Mayo College of Medicine, Rochester, Minnesota, United States of America; Virgen Macarena University Hospital, School of Medicine, University of Seville, Spain

## Abstract

DNA methylation plays a critical role in the regulation of gene expression, genomic DNA stability, cell proliferation, and malignant transformation. Common cellular features including fast tissue expansion, invasive growth, and active angiogenesis, have been noticed between placental development and tumorigenesis by many investigators. While the DNA hypomethylation and transcriptional activation of LINE-1 has been found to be a feature of tumorigenesis, it is not clear if similar changes could be involved in placental development. In this study, we assessed LINE-1 methylation in human placentas from different gestational ages and observed a significant decrease of LINE-1 methylation levels in third trimester placentas compared to first trimester placentas. Accompanying with this change is the significantly increased LINE-1 mRNA levels in third trimester placentas. Since no global DNA methylation change was detected between first and third trimesters, LINE-1 methylation changes appeared to be a specific epigenetic entity contributing to placental development. Indeed, further analyses showed that LINE-1 upregulation was correlated with higher levels of PCNA, suggesting a link between LINE-1 activation and fast proliferation of certain cellular components in third trimester placentas. Measurement of the DNMT1, DNMT3A, and DNMT3B expression found a significant reduction of DNMT3B between third and first trimesters, pointing to the possible involvement of this enzyme in the regulation of LINE-1 methylation. Taken together these results provided evidence for a dynamic temporal regulation of LINE-1 methylation and activation during placental development. These studies have laid a foundation for future investigation on the function of LINE-1 expression in human placenta under different patho-physiological conditions.

## Introduction

In eukaryotic cells, approximately 1% of human genome, predominantly at the cytosine residues in the CpG dinucleotide sequences, is subject to methylation modification [1], [2]. This modification transmits heritable information along somatic lineages, and exerts profound influences on the accurate control of gene expression, cellular differentiation and fetal development in mammals [3]–[5]. Gene- and tissue-specific DNA methylation patterns are established in early embryogenesis through a well-orchestrated demethylation and de novo re-methylation scheme. It has been well known that through its control of gene expression, DNA methylation is involved in the regulation of a variety of cell functions [1], [3], [6], [7].

While some CpG dinucleotides concentrate in short genomic regions or CpG islands, most CpG sites distribute in a sporadic pattern along the intergenic and intronic regions, particularly in some retroviral repeat sequences such as the long interspersed nuclear element (LINE-1), short interspersed nuclear element (SINE) and Sat2 [8]. Hypomethylation of these repeat sequences has been linked to pathological processes including tumorigenesis, abnormal placental function, birth defects, aging and other chronic diseases [9], [10].

LINE-1 is the largest component among various interspersed repeat sequences, constituting approximately 18%∼20% of human genome with up to 500 000 copies [11], [12]. Although the majority of LINE-1 s are 5′-truncated and transcriptionally inactive, about 150 full-length LINE-1 s with two intact open reading frames (ORF) (ORF1 and ORF2), and an additional 100 LINE-1 s with only intact ORF2, have been found to be transcriptionally active [13]. LINE-1 transcription was previously reported to be active mainly in germ line and transformed cells [14]-[17]. Intact LINE-1 is an autonomous retrotransposon capable of causing insertion mutations and genome rearrangements, while ORF2 might also contribute to the mobilization of non-autonomous sequences such as Alu and SVA elements [18]. *In vitro* experiments showed that the expression of full-length LINE-1 or ORF2 alone could induce DNA damage and apoptosis in cancer cells, human fibroblasts, and adult stem cells [19]–[21]. Recently, expression of LINE-1 has been detected in a variety of somatic tissues [19], [22], [23]. It has been reported that the LINE-1 expression might be related to cell proliferation and differentiation. Some studies indicated that disruption of endogenous LINE-1 expression resulted in reduced proliferation of cancer cells [24].

DNA methylation is considered a critical regulatory mechanism for the control of LINE-1 expression [12], [25]–[27]. The majority of CpG-rich LINE-1 promoters are usually methylated and silenced in normal tissues [22]-[23], [27]. Since LINE-1 sequences are widely distributed in human genome, its methylation levels have been used by some investigators as a surrogate for global methylation [28]. Hypomethylation of LINE-1 is frequently observed in malignant tissues and proposed to be a useful biomarker for the prediction and prognosis of cancers [9], [28].

It is believed by many investigators that placental organogenesis and tumorigenesis share some common features, including the fast cell proliferation, a lack of cell-cell contact inhibition, invasive growth, the capability for acquisition of a rich blood supply through aggressive angiogenesis, and the capability of escaping immune surveillance [29]–[32]. While some common molecular circuits and regulatory pathways have been identified between placenta and cancer, it is not clear how much similarity they may share on the epigenetic level. Genomic DNA of term placenta was reported to be globally hypomethylated compared to that of other somatic tissues [2], [33]. Elevated LINE-1 methylation levels has been described in placentas of partial hydatidiform moles, suggesting that LINE-1 methylation may involve in the mal-development of placenta [34]. LINE-1 expression was also detected in normal human placenta [19], [23], but its function for placenta development has not been determined. An intriguing question is, whether the placenta may undergo changes in LINE-1 methylation and expression pattern along the transition of pregnancy stages, just like what occurs during malignant transformation. In this study, we determine the patterns of LINE-1 methylation and transcription in first trimester (1N) and third trimester (3N) placentas, and explore their relationship with cell proliferation in human placenta. In addition, we measure the mRNA levels of various isoforms of DNA methyltranferases (DNMTs) and analyze the potential correlation between DNMT expression and LINE-1 methylation/expression. Such studies on the temporal changes of LINE-1 methylation/expression will help us to better understand the dynamic nature of epigenetic regulation along placental development.

## Materials and Methods

### Placenta tissue preparation

This study was approved by the Mayo Institutional Review Board and all the samples were collected at the Department of Obstetrics and Gynecology, Mayo Clinic, Rochester, Minnesota. 9 placentas of first trimester (1N; gestation age ranging from 7 to 12 weeks) and 13 placentas of third trimester (3N; gestational age ranging from 31 to 41 weeks) were collected with patients' written consents. Pregnancies associated with fetal malformation were excluded from the study. The 1N samples were obtained from unintended abortion, except one sample from a pregnancy terminated for chronic hypertension. The 3N placentas were collected after cesarean section, including one case with maternal diabetes and one with prenatal diagnosis of Intra Uterus Growth Restriction (IUGR). As described under Results, special attention has been paid to the potential impact of these three samples from abnormal pregnancy, and data are analyzed and documented in both ways of with or without these three samples. Placental specimens were collected following procedures as previously described [35]. Briefly, a piece of tissues in 2 cm^3^ size were dissected from the maternal side in the central part of placentas. Following repeated rinse with cold PBS and removal of visible vessels and connective tissues, the samples were stored at −70°C until DNA and RNA extraction.

### DNA extraction and Combined Bisulfite Restriction Analysis (COBRA) of LINE-1 methylation

Genomic DNA was extracted from placental tissues with the use of Qiagen DNA mini Kit (Qiagen, Valencia, CA, USA). DNA bisulfite modification was achieved with Qiagen EpiTect Bisulfite Kit (Qiagen, Valencia, CA, USA) following the protocol recommended by the manufacturer. Bisulfite-treated DNA was stored at -20°C for later experiments.

**Figure 1:**
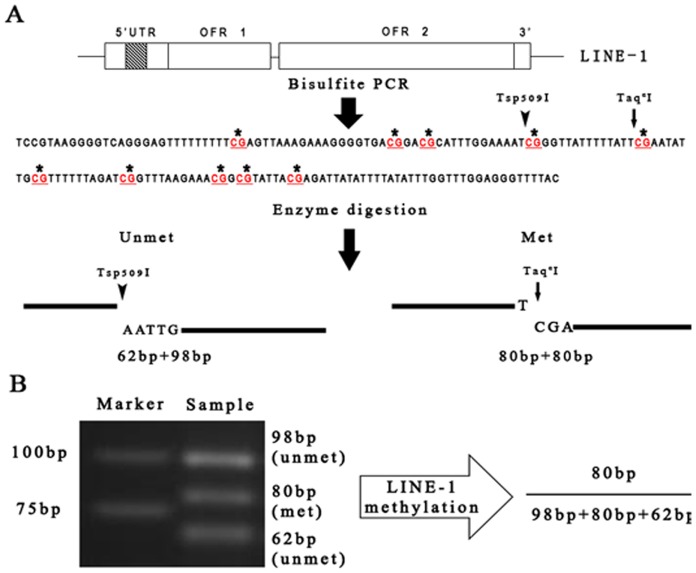
Schematic diagram of combined bisulfite restriction analysis (COBRA) of LINE-1. **A**. Intact LINE-1 is approximately 6 kb long, and comprised of 5′-untranslated region (5′UTR), two opening reading frames ORF1 and ORF2 and 3′UTR. After bisulfite conversion of genomic DNA, PCR was performed to amplify a region of 160 bp in 5′UTR (gray region with oblique lines) harboring 10 CpG sites (marked by asterisks). The site at 62 bp is cut by Tsp509I (target at ∧AATT) when CpG is unmethylated, yielding fragments of 62 bp and 98 bp (marked as unmet). The site at 80 bp is cut by Taq^α^I (target at T∧CGA) when CpG is methylated, yielding two fragments of 80 bp each (marked as met). **B**. After digestion with restriction enzymes, PCR products were separated by electrophoresis in 2.3% agarose gels. DNA bands were visualized and subject to densitometry analyses. The LINE-1 methylation level is calculated as the percentage of the intensity of Taq^α^I-positive fragment in total DNA.

The procedures of LINE-1 COBRA were illustrated in Figure 1. Using bisulfite modified DNA as template, a consensus sequence of 160 bp representing the LINE-1 5′UTR was amplified as previously described by Chalitchagorn [36] with some modifications. PCR was performed in 50 µl volume containing 1X Mg-free reaction buffer (ABI, Foster City, CA, USA), 2.0 mM of MgCl_2_, 1 µM of DNTP mix (Invitrogen, Carlsbad, CA, USA), 1 unit of Taq polymerase (ABI, Foster City, CA, USA), 10 pmol of the forward primer LINE-1F (5′-GYGTAAGGGGTTAGGGAGTTTTT-3′), 10 pmol of the reverse primer LINE-1R (5′- AACRTAAAACCCTCCRAACCAAATATAAA-3′) and 1 µl of bisulfite-converted DNA. Following the initial denature at 96°C for 5 min, 30 cycles of amplification were performed under the conditions: 96°C for 60 s, 56°C for 45 s and 72°C for 30 s. LINE-1 methylation levels were estimated by the methylation status of two CpG sites. The 160 bp PCR amplicon was digested in 10 µl reaction using 1 unit of Taq^α^I (New England Biolabs, Ipswich, MA, USA) and 4 units of Tsp509I (New England Biolabs, Ipswich, MA, USA) at 65°C for 3 hrs. DNA fragments were separated by electrophoresis using 2.3% agarose gel (Fermentas, Glen Burnie, MD, USA). The restriction enzyme Taq^α^I cut the DNA amplicon at site TCGA (at 80 bp of the PCR amplicon), yielding two DNA fragments of 80 bp each for methylated LINE-1, whereas Tsp509I recognizes the site AATTG (at 62 bp of the PCR amplicon), generating two DNA fragments of 62 bp and 98 bp, which represents the unmethylated LINE-1 (Figure 1A). Densitometry analysis was performed using the Bandscan software (Glyko, Novato, CA, USA). LINE-1 methylation index was calculated as a ratio of the intensity of methylated fragment in total DNA (methylated plus unmethylated) (Figure 1B). COBRA was performed thrice for each sample. The final results are presented as mean ± standard error.

### LINE-1 subcloning and sequencing

Following PCR amplification as described above, PCR products from five first or third trimester placentas, respectively, was subcloned with Perfect PCR Cloning Kit (5Prime, Gaithersburg, MD, USA) following the manufacturer's instructions. Individual colonies were picked and grown overnight in LB (Luria-Bertani) Broth medium. Plasmid DNA was purified with PerfectPrep Spin Mini Kit (5Prime, Gaithersburg, MD, USA). After confirmation by EcoR-I digestion and gel electrophoresis, the positive clones were sent to ACGT, INC. (Wheeling, IL, USA) for DNA sequencing.

### Immunostaining of 5-methylcytidine (5-mC) in placental tissues

Sections in 4 µM thickness were prepared from formalin-fixed, paraffin embedded placental tissues. The sections were deparaffinized and rehydrated by sequential treatment with Xylene (5 min×2), 100% ethanol (3 min×2), 95% ethanol (3 min×2), 80% ethanol (3 min×1), 70% ethanol (3 min×1), and distilled water (1 min×2). Antigen retrieval was achieved by 2 steps. Tissue slides were incubated in 10 mM Citrate Buffer (pH 6.0) at 90°C water bath for 10 min. The slides were cooled down at room temperature for 20 min, and then treated with 2 N hydrochloric acid (HCl) for 30 minutes at 37°C. Endogenous peroxidase activity was quenched with 3% hydrogen peroxide. After blocking with Tris Buffered Saline (TBS) containing 1% gelatin at room temperature for 1 hour, the sections were stained overnight with mouse anti-5-mC monoclonal antibody (AbD Serotec, Raleigh, NC, USA, 1/1800 dilution) at 4°C, followed by incubation for 1 hour at room temperature with the biotinylated horse anti-mouse secondary antibody (Vector Laboratories, Burlingame, CA, USA, 1/400 dilution). Between incubation steps the slides were washed twice with TBS containing 0.1% Tween-20. The biotin-horseradish peroxidase-mediated color development was carried out with the VECTASTAIN Elite ABC kits (Vector Laboratories, Burlingame, CA, USA) and Diaminobenzidine tetrahydrochloride (DAB) (Sigma-Aldrich, St Louis, MO, USA) following the manufacturer's recommendations. Hematoxylin counterstaining was subsequently performed to show the nuclei of cells. 5-mC staining images were captured and analyzed with a Carl Zeiss AxioVert 200M microscope imaging system (Carl Zeiss, Thornwood, NY, US). Each sample was scored according to the degree of intensity and proportion of positively stained cells: negative (no positive staining)  = 0, weakly positive (weakly positive staining within the nucleus)  = 1, positive (positive staining within the nucleus)  = 2, and strongly positive (strong staining signal within the nucleus)  = 3. The final score of one sample was calculated by adding together the scores from each intensity category (proportion of cells multiply the intensity levels).

### RNA isolation, cDNA synthesis, and quantitative real-time PCR

Total placental RNA was isolated with Trizol reagents (Invitrogen, Carlsbad, CA, USA) as previously described [37]. Reverse transcription was carried out with High Capacity RNA-to-cDNA Kit (ABI, Foster City, CA, USA) using 2 µg RNA in 20 µl volume reactions. The 20 µl cDNA product was diluted into 100 µl for later use in real-time PCR. The mRNA expression levels of glyceraldehyde phosphate dehydrogenase (GAPDH), proliferating cell nuclear antigen (PCNA), LINE-1, DNA methyltransferases 1 (DNMT1), DNMT3A, and DNMT3B, were measured with real-time PCR (ABI 7900 Real-Time PCR System) in 12 µl reactions containing 6 µl of SYBR Green PCR Master Mix 2X (Applied Biosystems, Foster City, CA, USA), 1 µl of forward primer, 1 µl of reverse primer, 3 µl of H_2_O and 1 µl of diluted cDNA template under the conditions: initial denature, 95°C for 10 min, followed by 40 cycles of denature at 95°C for 30 sec, annealing at 54.5°C for 30 sec, and extension at 60°C for 1 min. The designations and sequences of the primers, and the sizes of resultant amplicons are summarized in Table 1. Following extensive optimization, specific amplification, as indicated by the single band pattern with predicted size, was achieved for each reaction (Figure S1). The threshold cycles (C_T_) were determined in triplicate reactions, and the data from target genes were standardized by the results of GAPDH or PCNA as internal reference genes. The standardization and comparison was based on the 2^-△△CT^ relative quantification method [38]. The relative mRNA levels were presented as mean ± standard errors (M±SE).

**Table 1:** Primers used in real-time quantitative PCR.

Gene	Primer sequences	Amplicon size
**GAPDH**	F 5'-AGGTGAAGGTCGGAGTCA-3'	98 bp
	B 5'-GGTCATTGATGGCAACAA-3'	
**PCNA**	F 5′-AGGAAGCTGTTACCATAGAGA-3′	135 bp
	B 5′-ACAACAAGGGGTACATCTGC-3′	
**DNMT1**	F 5'-AGGAGGGCTACCTGGCTAAA-3'	85 bp
	B 5'-CACTTCCCGGTTGTAAGCAT-3'	
**DNMT3a**	F 5'-TATTGATGAGCGCACAAGAGAGC-3'	115 bp
	B 5'-GGGTGTTCCAGGGTAACATTGAG-3'	
**DNMT3b**	F 5'-GGCAAGTTCTCCGAGGTCTCTG-3'	112 bp
	B 5'-TGGTACATGGCTTTTCGATAGGA-3'	
**LINE-1**	F 5'- TGTCCAAAACACCAAAAGCA -3'	113 bp
	B 5'- TTGCCTGTTCACTCTGATGG -3'	

F: forward, B: backward.

### Statistical analysis

All statistical treatments were performed with the SPSS statistical package 13.0 (SPSS, Chicago, IL). The comparison of methylation and mRNA levels between 1N and 3N placentas, was carried out with the use of Student's *t* test. The difference of 5-mC scores between groups was calculated with Mann-whitney U test. Correlation analysis was performed with rank correlation. Spearman's rank correlation coefficient (*r_s_*) and statistical significance (*P*<0.05) were presented in the tables and charts.

## Results

### LINE-1 methylation in first and third trimester placentas

**Figure 2:**
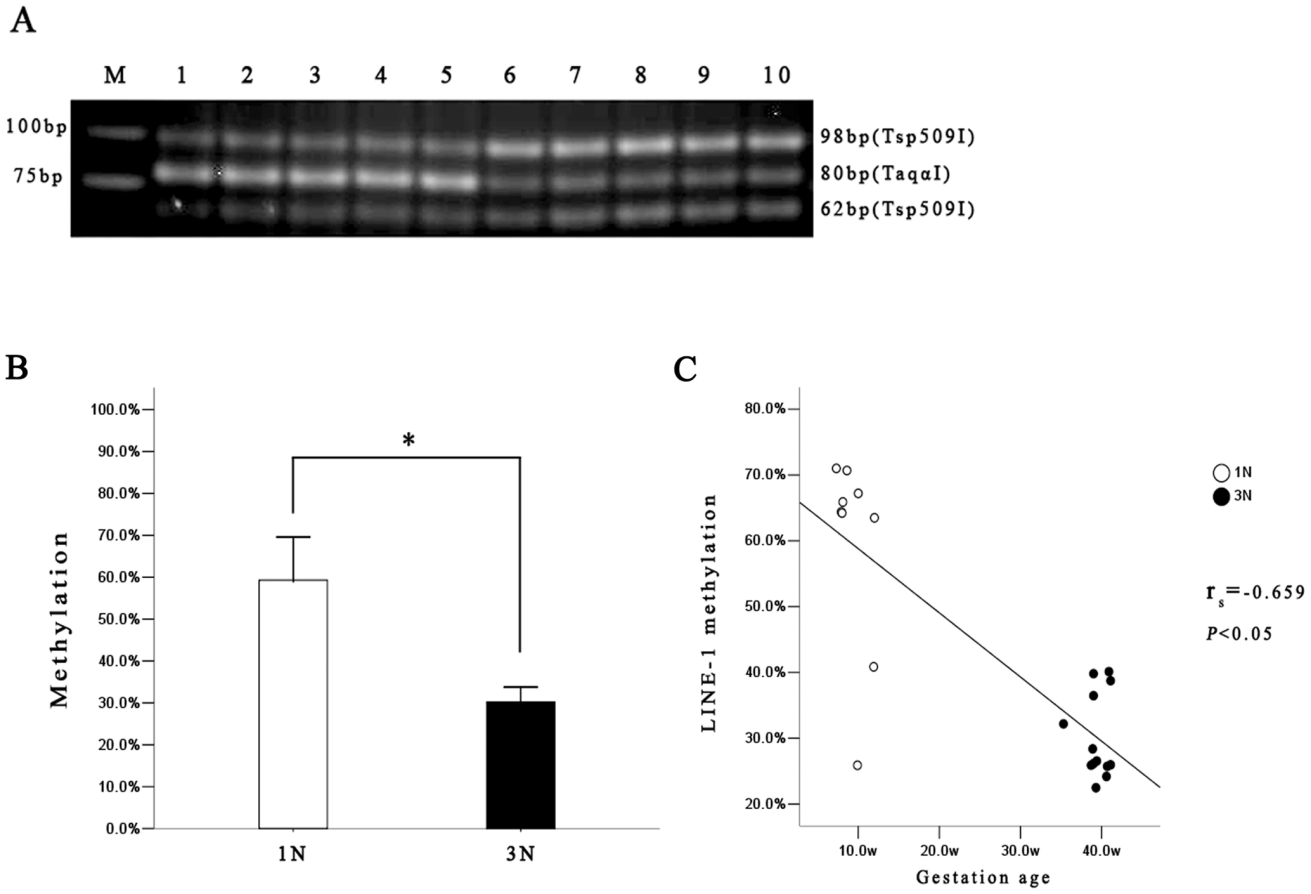
Assessment of LINE-1 methylation of first (1N) and third trimester (3N) placentas by COBRA. **A**. A representative gel picture of LINE-1 COBRA showing the DNA bands for methylated (80 bp) and unmethylated LINE-1(98 bp and 62 bp). Lanes 1–5: 1N placental samples; Lane 6–10: 3N placental samples. **B**. The results of densitometry analyses on LINE-1 methylation. LINE-1 is hypomethylated in 3N placentas (Mean: 30.2%) relative to 1N placentas (Mean: 59.3%). * *P*<0.05. **C**. Inverse correlation between LINE-1 methylation levels with gestation age. W: weeks.

LINE-1 DNA methylation levels at the 5′UTR were determined in the first (1N) and third trimester (3N) human placentas with the use of COBRA method (Figure 2A). We noticed a relatively large variation among the first trimester placentas. In 7 out of 9 placentas (7/9), LINE-1 methylation levels were higher than 60.0% of the total DNA. But in two samples, the methylation levels were strikingly lower, at 25.9% (12 weeks) and 40.8% (10 weeks) of total DNA, respectively (Figure S2). The variations were not associated with the gestation age, and review of the clinical data found no remarkable clinical manifestations in the two patients. Despite this intra-group variation, comparison of the two group representing different gestation ages indicated a significant change in LINE-1 methylation. The average LINE-1 methylation level of 3N placentas was 30.2%±6.4%, significantly lower than that of 1N placentas (59.3%±15.4%) (*P*<0.001) (Figure 2B). Despite the shortage of data representing the second trimester samples, we performed a tentative analysis on the changes of LINE-1 methylation levels along pregnancy. While the results pointed to a negative correlation between LINE-1 methylation levels and gestation ages in all placental samples (*r_s_* = −0.659, *P*<0.05) (Figure 2C), as discussed later, the result was not conclusive regarding the pattern and time of the LINE-1 methylation change. On the other side, as shown in Supplemental Data, intra-group correlation analysis in either the first (Figure S3A) or third (Figure S3B) trimester placentas found no significant association between LINE-1 methylation levels and gestation ages (*P*>0.05), suggesting the methylation modification occurred likely in second trimester.

**Figure 3:**
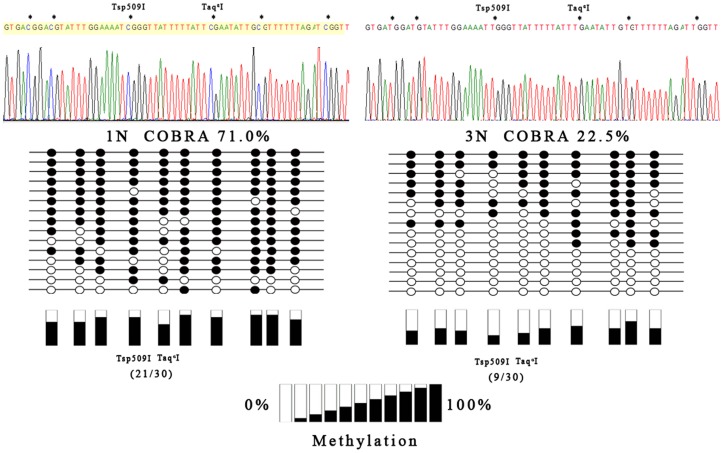
Results of LINE-1 bisulfite sequencing. Bisulfite converted DNA sequences and a typical sequencing result is shown at the top panel. Asterisks indicate the CpG sites. The cutting sites for Tsp509I and Taq^α^I were marked. 15 clones representing five first trimester (1N, left penal) placentas (3 clones each) and 15 clones representing five third trimester (3N, right panel) placentas (3 clones each) were sequenced. The solid and open circles represent the methylated CpG dinucleotides and unmethylated CpG, respectively. Methylation levels of each CpG sites were illustrated at the bottom panel. The CpG sites of LINE-1 in 3N placentas are generally hypomethylated relative to 1N placentas.

To further confirm the results of COBRA, LINE-1 5′UTR region was PCR amplified using bisulfate converted DNA template, subcloned into a plasmid vector provided by the Perfect PCR Cloning Kit, and subject to direct sequencing. Figure 3 summarizes the sequencing results from representative samples of 1N (5 samples, 3 clones from each sample) and 3N (5 samples, 3 clones from each sample) groups. The results confirmed that the DNA fragment represents the 5′UTR of LINE-1 sequences (GenBank: L19092.1), and complete bisulfite conversion of unmethylated cytosines has been achieved based on the changes to thymidine of the cytosine (unmethylated) in non-CpG dinucleotides contexts. Compared to 1N samples, the LINE-1 CpG sites from 3N placentas are significantly hypomethylated (22.5% vs.71.0%, *P*<0.05). For the CpG sites examined by COBRA, the results from direct sequencing showed average methylation levels of 70.0% (21/30) and 30.0% (9/30), respectively in 1N and in 3N samples (Figure 3). Thus the sequencing results are in general agreement with those of COBRA. Taking together, these results indicated that LINE-1 was relatively hypomethylated in 3N placentas compared to 1N placentas.

**Figure 4:**
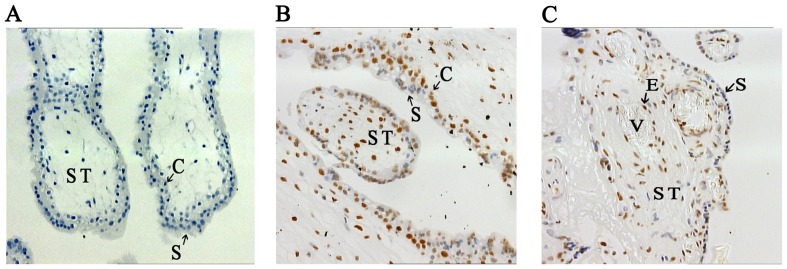
5-Methylcytidine (5-mC) immunostaining of placental samples. Tissue sections from paraffin-embedded placental tissues were treated with primary (Mouse anti 5-mC, concentration: 1/800), secondary (horse anti-mouse, concentration: 1/400) antibodies. Color development was performed with immunoperoxidase system. The 5-Methylcytidin-positive cells were stained brown, while negative cells displayed blue color of hematoxylin counterstaining. **A**. The negative control (1N) (20×10 magnitude) without primary antibody. **B**. 5-mC immunostaining of 1N placenta (20×10 magnitude). **C**. 5-mC immunostaining of 3N placenta (20×10 magnitude). Compared to 1N placenta, thinner lay of syncytium was observed in 3N placenta. Syncytiotrophoblasts (S) were stained relatively lighter than cytotrophoblast (C), stromal cells (ST), and epithelial (E) cells. V, blood vessels. Overall, similar intensity of 5-mC staining was observed 1N and 3N placentas.

LINE-1 repetitive element constitutes a substantial part of the human genome, and the changes in LINE-1 methylation may reflect a genome wide, global level of alteration. Therefore, we performed 5-mC immunostaining to evaluate the global DNA methylation status. Generally, different types of cells in placental tissues exhibited diverged DNA methylation levels in both 1N and 3N placentas. Syncytiotrophoblasts displayed relatively lower DNA methylation levels than stromal cells, cytotrophoblasts and vascular endothelial cells (Figure 4 and Table 2). The diverged staining densities among different cells verified the specificity of the immunohistochemistry signals. Surprisingly, repeated immunostaining in four pairs of 1N and 3N placental samples detected no significant difference in the global DNA methylation between the two groups (Immunostaining scoring, 1N median vs. 3N median, 1.16 vs. 1.04, *P* = 0.127). Thus, the discordance in LINE-1 and global methylation separated LINE-1 into a distinct entity for epigenetic regulation in placenta.

**Table 2:** 5-mC staining scores between the groups.

	Syncytiotrophoblast	Other cells*	P value
**1N**	**0.77**	**1.64**	**<0.05**
**3N**	**0.54**	**1.51**	**<0.05**

Mann-whitney U test, Data are shown as the Median; Other cells*: stromal cells, cytotrophoblasts and vascular endothelial cells.

### LINE-1 expression in placentas and its correlation with LINE-1 methylation

**Figure 5:**
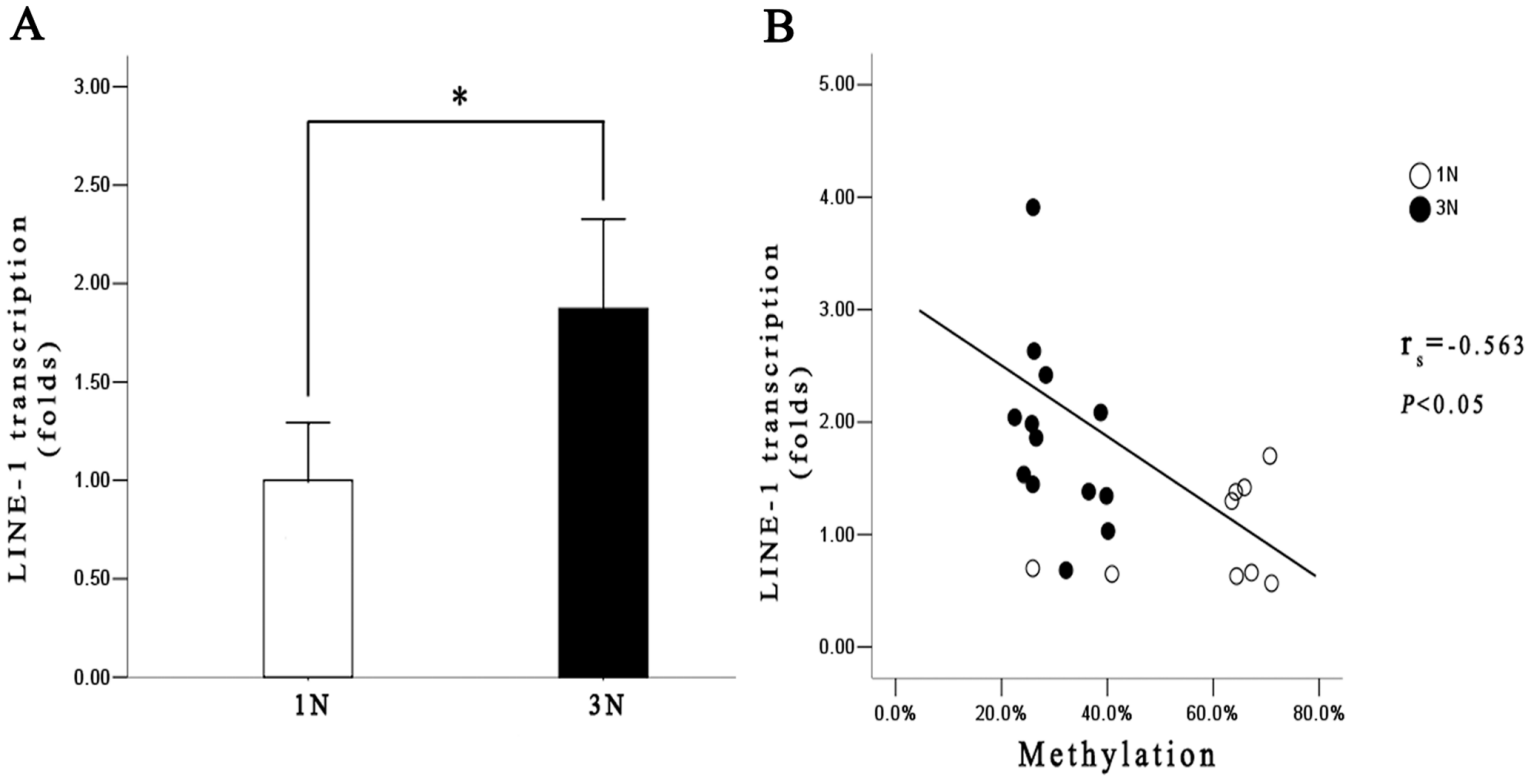
LINE-1 mRNA levels in first trimester (1N) and third trimester (3N) placentas and their correlation with methylation. **A**. Average LINE-1 mRNA levels were 1.9 folds higher in 3N placentas compared to 1N placentas (*P*<0.05). **B**. Inverse correlation of LINE-1 mRNA levels with methylation levels in 1N and 3N placentas (Spearman correlation, *r_s_* = −0.563, *P*<0.05)

The findings on the alterations of DNA methylation in the LINE-1 5′UTR prompted us to further examine the LINE-1 mRNA expression. We determined LINE-1 mRNA levels using real-time PCR, and found that average LINE-1 mRNA level in 3N placentas is 1.9 times higher than that of 1N placenta (*P*<0.05) (Figure 5A). Moreover, the increased mRNA level is closely correlated with the decreased methylation in LINE-1 5′UTR (*r_s_* = −0.563, *P*<0.05) (Figure 5B). These results suggested that the 5′UTR methylation of LINE-1 may participate in the regulation of LINE-1 transcription during the development of human placenta.

### The status of cell proliferation in placenta and its relationship with LINE-1 methylation and transcription

**Figure 6:**
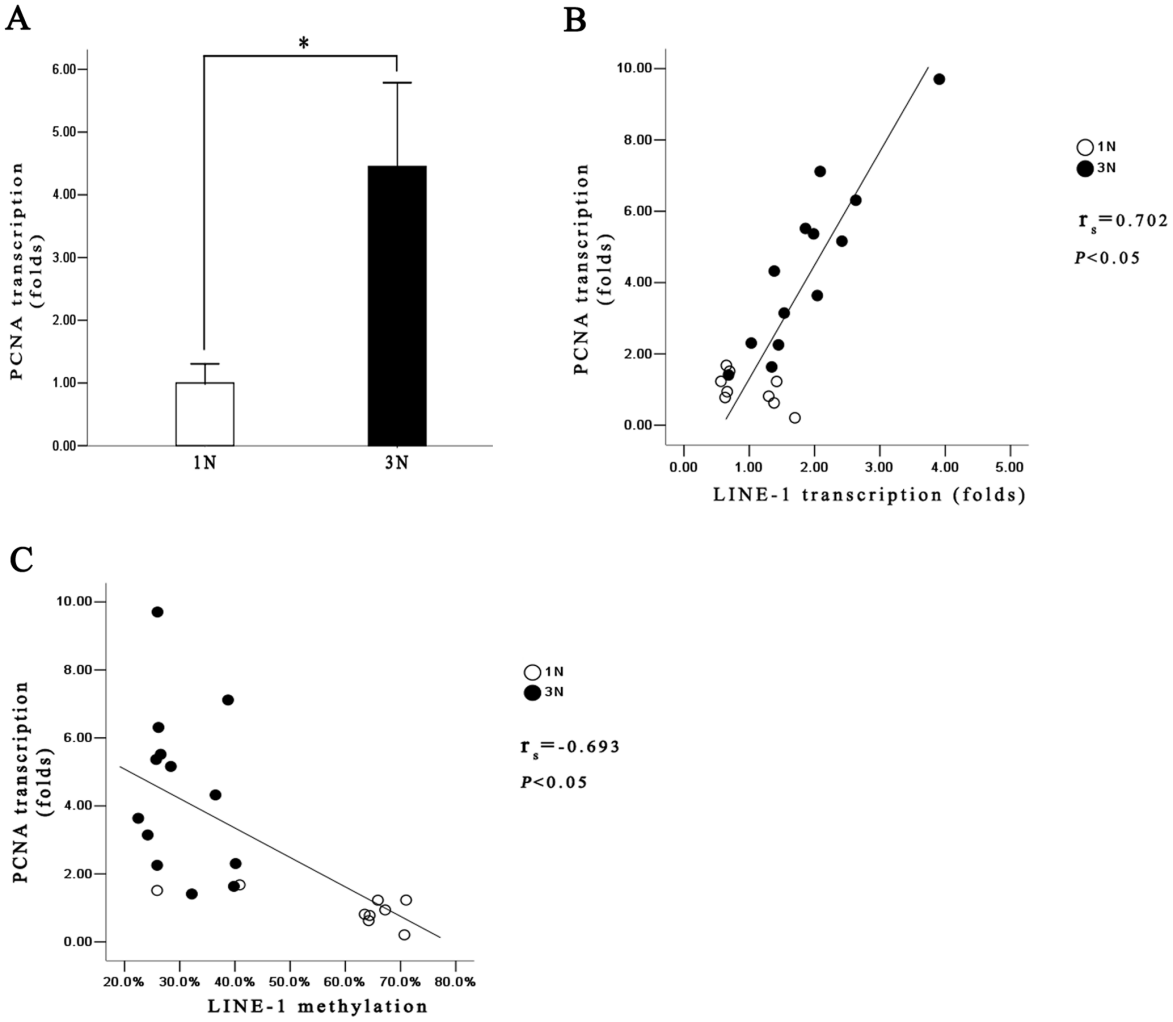
PCNA mRNA levels in first trimester (1N) and third trimester (3N) placentas, and their association with LINE-1 mRNA levels and methylation status. **A**. PCNA mRNA levels of 3N placentas were significantly higher than those of 1N placentas (4.5 folds, *P*<0.05). **B**. PCNA mRNA levels in 1N and 3N placentas were positively correlated with those of LINE-1 mRNA levels (Spearman correlation, *r_s_* = 0.702, *P*<0.05). C. PCNA mRNA levels in 1N and 3N placentas were negatively correlated with those of LINE-1 methylation (Spearman correlation, *r_s_* = −0.693, *P*<0.05).

It was previously reported that LINE-1 expression is related to cell proliferation and differentiation in cancer cells [24]. We subsequently investigated if LINE-1 methylation and transcription could be associated with placental cell proliferation. We used PCNA as a marker for cell proliferation and measured its mRNA level in 1N and 3N placentas. The results showed that the PCNA mRNA level in 3N placenta was 4.5-fold higher than that of 1N placenta (*P*<0.05), suggesting a relatively more active cell proliferation in 3N placenta (Figure 6A). Regression analysis among all the placental samples indicated that the elevated cell proliferation in 3N placenta was positively correlated with the elevated LINE-1 transcription (*r_s_* = 0.702, *P*<0.05) (Figure 6B). In addition, the PCNA mRNA levels were also closely correlated with the LINE-1 methylation levels (*r_s_* = −0.693, *P*<0.05) (Figure 6C). These results pointed to a possible association of LINE-1 demethylation/activation with increased cell proliferation along placental development. The potential underlying mechanisms will be explained under Discussion.

### DNMTs expression in first and third trimester placentas and its relationship to LINE-1 methylation

In mammalian cells genomic DNA methylation is carried out by DNA methyltransferases, and some studies have shown that methylation patterns in several types of cancers were related to the expression levels of DNMTs [39]–[41]. To examine whether the LINE-1 methyaltion alteration in human placenta may be related to changes in DNMT expression, we perform real-time PCR to compare the mRNA levels of DNMT1, DNMT3A and DNMT3B between 1N and 3N placentas. First, when GAPDH mRNA levels were used as internal references for a “static” analysis of DNMT mRNA expression levels, mRNA levels of both DNMT1_GAPDH_ and DNMT3B_GAPDH_ appeared to be elevated in 3N group compared to 1N group. While DNMT3B_GAPDH_ level in 3N group was 1.5 fold of that in the 1N group, the change did not reach a statistically significant level. These results seemed to suggest that during the placental development along the gestation stage, placentas express increased levels of DNMT1 and DNMT3B.

DNMTs have been reported to be intrinsically related to cell cycle, and the rapid growing cells and tissue tend to express higher levels of DNMTs [42]. Indeed, our results of correlation analysis confirmed this relationship. DNMTs mRNA levels standardized by those of GAPDH are significantly correlated to the PCNA mRNA levels (Table 3).

**Table 3:** Correlations between DNMTs and PCNA.

	DNMT1_GAPDH_	DNMT3a_GAPDH_	DNMT3b_GAPDH_
**PCNA**	0.674*	0.819*	0.447*

*Spearman's correlation, *P*<0.05.

By the same thought on the close association of DNMT expression with cell proliferation, in order to dynamically evaluate the expression levels of DNMTs and their contribution to DNA methylation, many investigators used PCNA mRNA levels as internal reference gene. We accordingly performed such analyses by using PCNA mRNA levels to standardize the DNMTs mRNA levels. The results (Table 4) indicated that DNMT1 mRNA levels relative to PCNA (DNMT1_PCNA_) in 3N placentas were 30% higher than those of 1N placentas, but no correlation with LINE-1 methylation was observed (*r_s_* = 0.205, *P*>0.05). The mRNA levels of *de novo* methyltransferases, DNMT3A_PCNA_ and DNMT3B_PCNA_, were up-regulated and down-regulated in 3N placentas (*P*<0.05), respectively, with significant but opposite correlations with LINE-1 methylation. Specifically, the DNMT3A_PCNA_ mRNA levels appeared to be inversely correlated with LINE-1 methylation levels (*r_s_* = −0.683, *P*<0.05; figure not shown), while DNMT3B_PCNA_ mRNA levels displayed a positive correlation with LINE-1 methylation levels (*r_s_* = 0.508, *P*<0.05; figure not shown). Thus, following PCNA standardization, the data showed lower expression levels of DNMT1 and DNMT3B of 3N placentas than those of 1N placentas. It is not clear at this time if this reduction might be involved in the decreased LINE-1 methylation levels along the placenta maturation. On the other hand, the inverse correlation between DNMT3A and LINE-1 methylation is a puzzling dilemma that is difficult to be comprehended based on the current knowledge.

**Table 4:** DNMTs mRNA levels in first trimester (1N) and third trimester (3N) placentas.

	Reference gene	1N#(*M±2SE*)	3N(*M±2SE*)
**DNMT1**	GAPDH	1.00±0.42	4.66±2.01^*^
	PCNA	1.00±0.20	0.70±0.13
**DNMT3a**	GAPDH	1.00±0.58	15.20±6.91^*^
	PCNA	1.00±0.29	2.43±0.67^*^
**DNMT3b**	GAPDH	1.00±0.40	1.49±0.61
	PCNA	1.00±0.30	0.24±0.09^*^

# The means of DNMTs mRNA levels in first trimester (1N) placentas were set at 1, and DNMTs mRNA levels from third trimester (3N) placentas were presented as the relative folds to those of 1N group. Asterisks indicate that the difference between 1N and 3N placentas reached statistical significance (*P*<0.05). M: mean, SE: standard error.

### The influence of maternal and fetal complications on the data analysis

In this study, 3 placental samples were collected from cases with maternal or fetal complications (1N: one with maternal hypertension; 3N: one with maternal diabetes and one with IUGR). To test whether the data from these complications would affect the results, we re-performed all the above analyses by excluding the 3 samples from both groups and found no impact on the statistical results. Thus, inclusion of these samples does not affect our overall conclusions.

## Discussion

The loss of methylation and activated transcription in LINE-1 has been detected in a variety of cancers and thought to be a characteristic alteration in neoplasia [9], [16], [25], [28], [36], [43]. Placenta is considered a ‘pseudo-malignant’ tissue that shares several important features with cancers. Our data showed that LINE-1 methylation status undergoes dramatic transition when pregnancy proceeds. Compared to first trimester placentas, LINE-1 methylation was significantly decreased in third trimester samples. The transition is also demonstrated by an inverse correlation with gestation age, suggesting that LINE-1 demethylation and the accompanying overexpression may contribute to placenta development. Thus, the transition of LINE-1 methylation in placenta appears to be reminiscent of the observation in cancer development, suggesting that similar epigenetic regulation mechanisms may exist in the two processes.

It should be pointed out that due to the difficulty to obtain mid-term pregnancy placental samples, the available specimens only covered 7 to 12 weeks for the first trimester, and 35 to 41weeks for the third trimester, leaving a large gap between the two groups. This technical discontinuation in the available data certainly compromised the efficiency of correlation analysis and the convincing power of the result (Fig 2C). Therefore, we must take precautions when considering the features of LINE-1 methylation alteration along pregnancy. When data from the two trimesters were analyzed separately, none of them showed correlation between LINE-1 methylation and pregnancy ages (Fig S3), indicating an absence of significant alteration in the first or third trimester. When the first and third trimester placentas were analyzed in a combined manner, the LINE-1 methylation appeared to be decreased along pregnancy. But as mentioned above, because of the lack of data representing mid-term pregnancy, this result could not tell us the pattern (e.g., sudden or gradual) or the exact time, of the observed alteration that most likely occurred in the second trimester. Further investigation focusing on second trimester may help to clarify the pattern and timeline of this epigenetic alteration during pregnancy.

The significant decrease of LINE-1 methylation level along gestation is in a sharp contrast to the trend of global methylation levels that has been shown to be either increased [44] or unchanged (our results). LINE-1 comprises a substantial portion of the human genome, and its methylation level was thought to be representative of global DNA methylation [28] in non-placental tissues. However, placenta is a highly specialized extraembryonic organ characterized by a fast growth, hypoxic condition, and a relatively short “life”. The segregation of LINE-1 methylation patter from that of global methylation suggests that LINE-1 may represent a special entity for epigenetic regulation in placenta. The correspondent increase in LINE-1 mRNA expression in the third trimester (Fig-5A, Fig-5B) provides a corroborate evidence for this thought. Taken together, these results indicated that the decreasing LINE-1 expression may represent an element of a constitutive program directing placental development.

In cancer cells, interference of LINE-1 expression by siRNA or inhibition of the LINE-1-produced reverse transcriptase resulted in inhibition of cell proliferation [21], [24], [45]–[47]. Similarly, LINE-1 expression may be essential for cell proliferation in placenta. Human placenta is composed of cytotrophoblasts, syncytiotrophoblasts, stromal cells, vascular endothelial cells, and smooth muscle cells. Although the renewal of trophoblasts was reported to be more frequent in early placentas than term placentas [48], angiogenesis becomes increasingly more active from late first trimester and this trend continues to the term [49]. Thus increased LINE-1 expression may be attributed to the active angiogenesis in 3N placentas. Alternatively, LINE-1 expression might serve as a negative modulator to restrict cell proliferation of trophoblast linage in the third trimester. Indeed, in term placentas many cytotrophoblasts fuse to form syncytiotrophoblasts which have reached a terminal differentiation stage, and lost the mitotic activity. It is noteworthy that overexpression of full-length LINE-1 or only ORF2 in human fibroblasts and adult stem cells led to a senescence-like phenotype [19]. The progressively increased LINE-1 transcription along with the gestation age may be related to increased cell fusion. This view is consistent with the previous finding that LINE-1 expression is suppressed in cytotrophoblasts [23]. Further investigation is required to understand the exact role of LINE-1 demethylation and increased expression in third trimester placentas.

Data for gestational change in placental global DNA methylation appears to be limited and controversial. Fuke et al applied HPLC technique to measure the methylated cytosine levels in placentas of different gestation ages. A gradual increase in global methylation along gestation was observed [50]. However, no background information, such as the presence of maternal complications, gender of the fetus, conditions of abortion (spontaneous or induced), and the position for placenta sampling, is available, making it difficult to compare to other studies. More recently Chavan-Gautam et al [44] used methylamp™ technique to assess methylation levels in term and preterm placentas and reported a positive association between methylation and gestational age. Grazul-Bilska et al [51] investigated the sheep placentas collected from 16 to 30 days of gestation age (corresponding to first trimester in human) and found a steadily reduced methylation levels along placental development when DNA dot blot technique was used. However, immunohistochemistry using the 5-mC antibody showed no significant change in global methylation, which was similar to the results of current study. The discrepancy in the published data may be partially attributed to the application of different techniques. Also, it is important to point out that neither preterm nor spontaneously aborted placentas are reliable sources of samples due to the potential interference by genetic defects often harbored in these placental tissues. More studies using placental tissues with better defined conditions, e.g., by exclusion of genetic defects and IUGR/preeclampsia, as well as the control of health-, nutrition-, and social-related factors, will be required to solve this controversy.

DNMTs are the enzymes responsible for the establishment and maintenance of genomic DNA methylation patterns. We found that DNMTs are differentially expressed in 1N and 3N placentas in an isoform-specific manner. Overall, the expression of DNMT1, DNMT3A and DNMT3B significantly elevate as gestation age advances. The elevation appears to coincide with the increased cell proliferation as indicated by the levels of PCNA. This result appears to be in agreement with the previous observation that fast growing cells tend to express high levels of DNMT. Presumably, the replicating cells express higher levels of DNMTs to maintain the methylation patterns in nascent cells [42]. However, when standardized by PCNA, a trend for decreased DNMT1 and DNMT3B mRNA levels (DNMT1_PCNA_ and DNMT3B_PCNA_) along placental development were observed, suggesting that despite their increase in absolute amount, there might be a relative deficiency for these enzymes in the context of fast cell proliferation. Intriguingly, an opposite trend of change was detected in DNMT3A mRNA levels when either GAPDH or PCNA was used as an internal reference. Previous studies have shown that while DNMT3A and DNMT3B are capable of modifying the hemimethylated DNA, the two enzymes have shown target preference for genomic DNA methylation. DNMT3B prefers certain repetitive sequences as methylation targets, whereas DNMT3A has a wider range of targets, including the non-CpG sequences. The divergent DNMT3A_PCNA_ and DNMT3B_PCNA_ changes along the placenta maturation seems point to their specific roles in DNA methylation. The strong positive correlation between DNMT3B_PCNA_ and LINE-1 methylation seems suggested that this enzyme may likely to play a role in LINE-1 methylation. It should be pointed out that as an initial investigation effort in this area, the current study on the correlation between LINE-1 methylation and DNMT expression remain largely descriptive and qualitative. DNA methylation is subject to stringent and complex regulations by multiple factors and pathways. More quantitative and mechanistic studies are required for a better understanding on the exact role(s) of DNMTs for LINE-1 methylation.

In conclusion, we have conducted an extensive correlation analysis among LINE-1 methylation and expression, global DNA methylation, DNMTs expression, and cell proliferation. The significant decrease in LINE-1 methylation along gestation age revealed an epigenetic feature that is shared between placental development and cancer progression. Moreover, the study has for the first time suggests that DNMT3B is likely involved in LINE-1 methylation. These observations depict a dynamic feature of epigenetic regulation in placenta, and paved the way for future mechanistic studies on the regulation and function of LINE-1 in human placenta under different patho-physiological conditions.

## Supporting Information

Figure S1 The specificity of real-time PCR. Products from real-time PCR were resolved in 2% agarose gel and DNA bands were visualized by ethidium bromide staining. The single band patterns with the expected sizes of real-time PCR products indicated that specific amplification was achieved in the real-time PCR, and the data from real-time PCR was reliable. M: marker.
(TIF)

Figure S2 Results of COBRA on LINE-1 methylation. LINE-1 is hypomethylated in 3N placentas (Mean: 30.2%) relative to 1N placentas (Mean: 59.3%). * *P*<0.05. Open circle (○): placental samples of 1N; Open triangle (△): placental samples of 3N. Note the two samples of 1N groups with substantial lower levels of LINE-1 methylation.
(TIF)

Figure S3 Spearman correlation analyses showed that no significant correlation between LINE-1 methylation levels and gestation ages were detected in either 1N (A) or 3N (B) placentas when the two groups were analyzed separately.
(TIF)
