# Supplementary figures and images for: Transition of LINE-1 DNA Methylation Status and Altered Expression in First and Third Trimester Placentas
Source: PLoS One. 2014 May 12;9(5):e96994. doi: 10.1371/journal.pone.0096994 (PMC4018393; doi:10.1371/journal.pone.0096994)

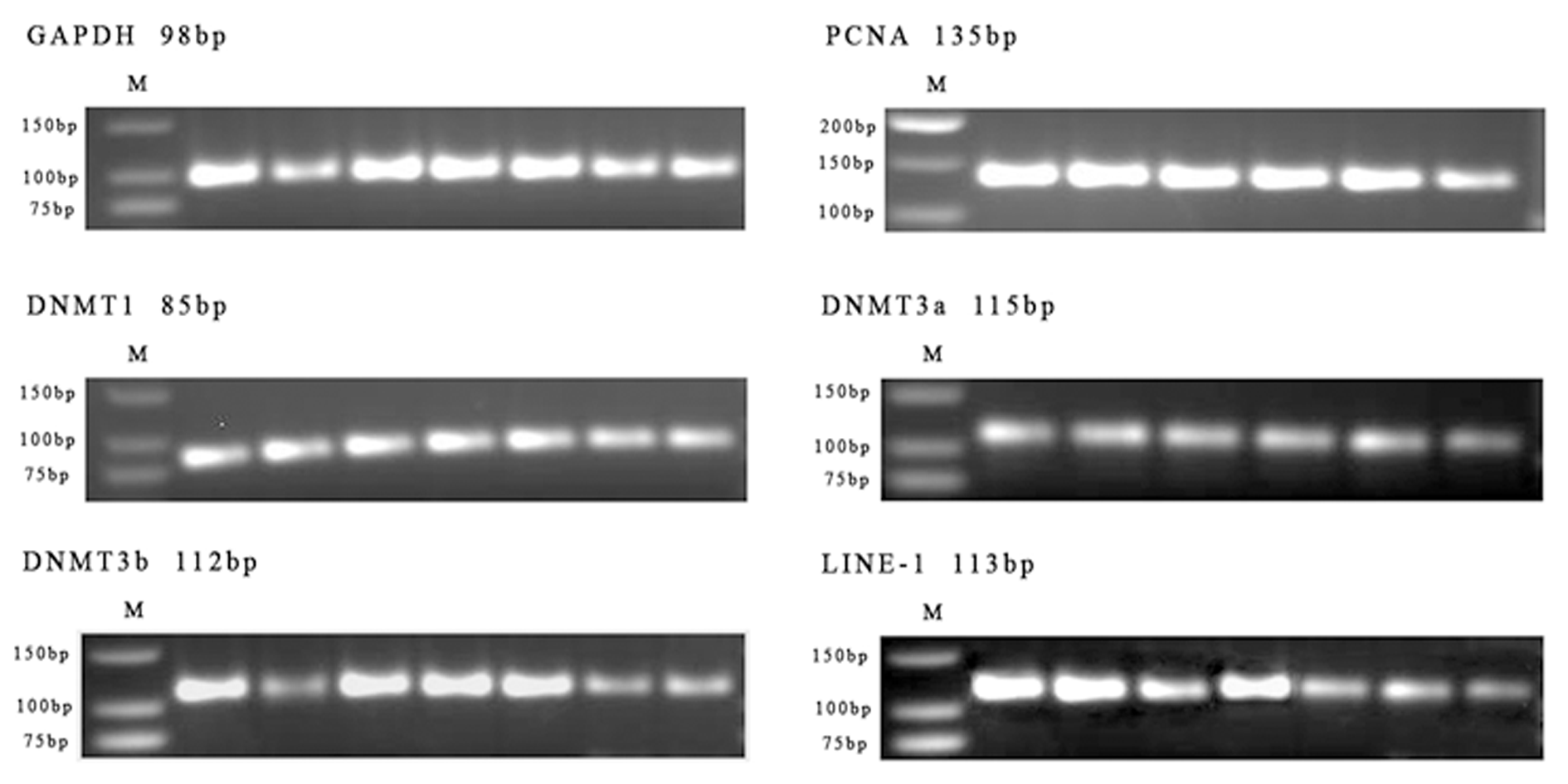

Supplement: Figure S1 — The specificity of real-time PCR. Products from real-time PCR were resolved in 2% agarose gel and DNA bands were visualized by ethidium bromide staining. The single band patterns with the expected sizes of real-time PCR products indicated that specific amplification was achieved in the real-time PCR, and the data from real-time PCR was reliable. M: marker. (TIF) [file pone.0096994.s001.tif]

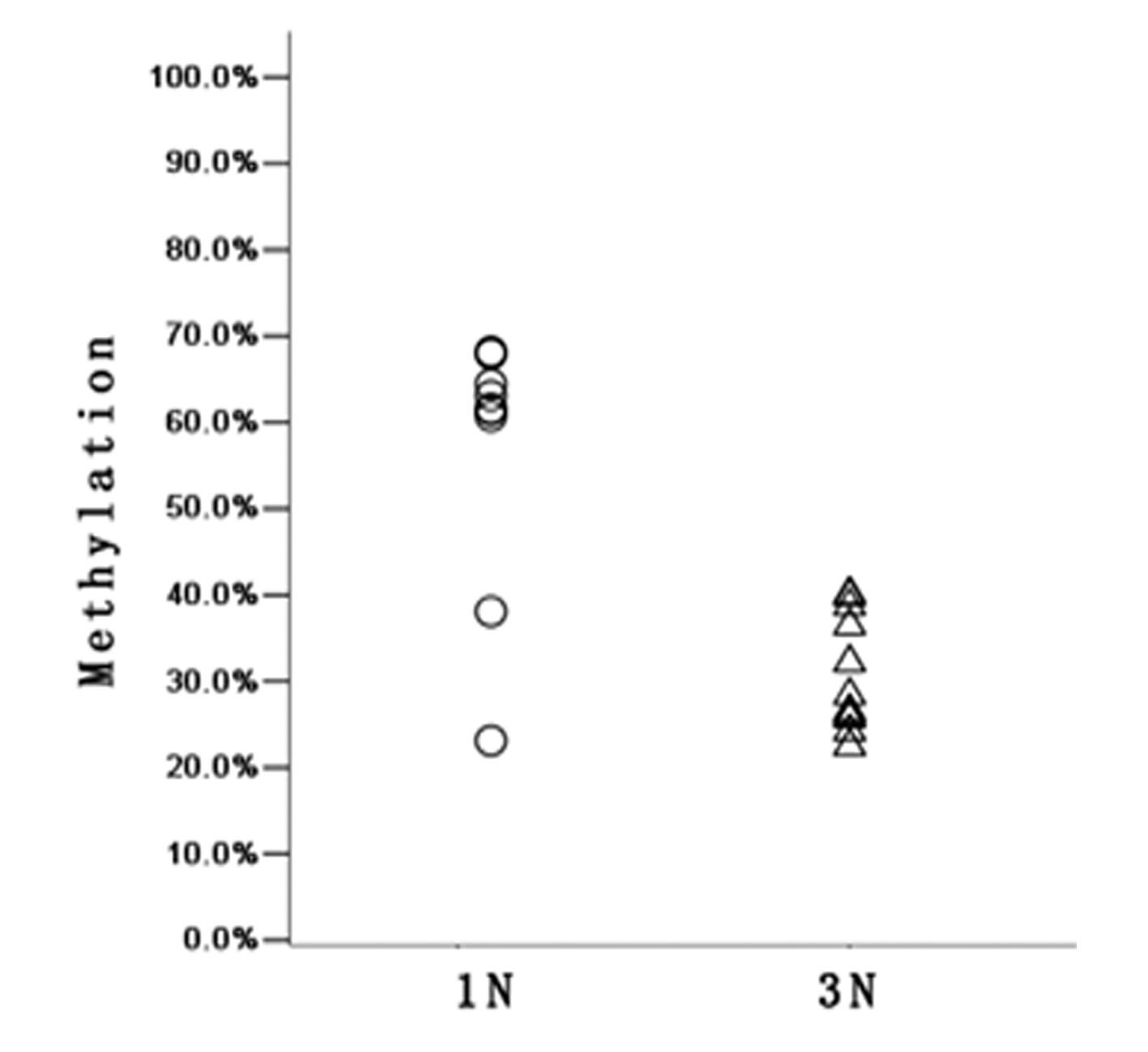

Supplement: Figure S2 — Results of COBRA on LINE-1 methylation. LINE-1 is hypomethylated in 3N placentas (Mean: 30.2%) relative to 1N placentas (Mean: 59.3%). * P<0.05. Open circle (○): placental samples of 1N; Open triangle (△): placental samples of 3N. Note the two samples of 1N groups with substantial lower levels of LINE-1 methylation. (TIF) [file pone.0096994.s002.tif]

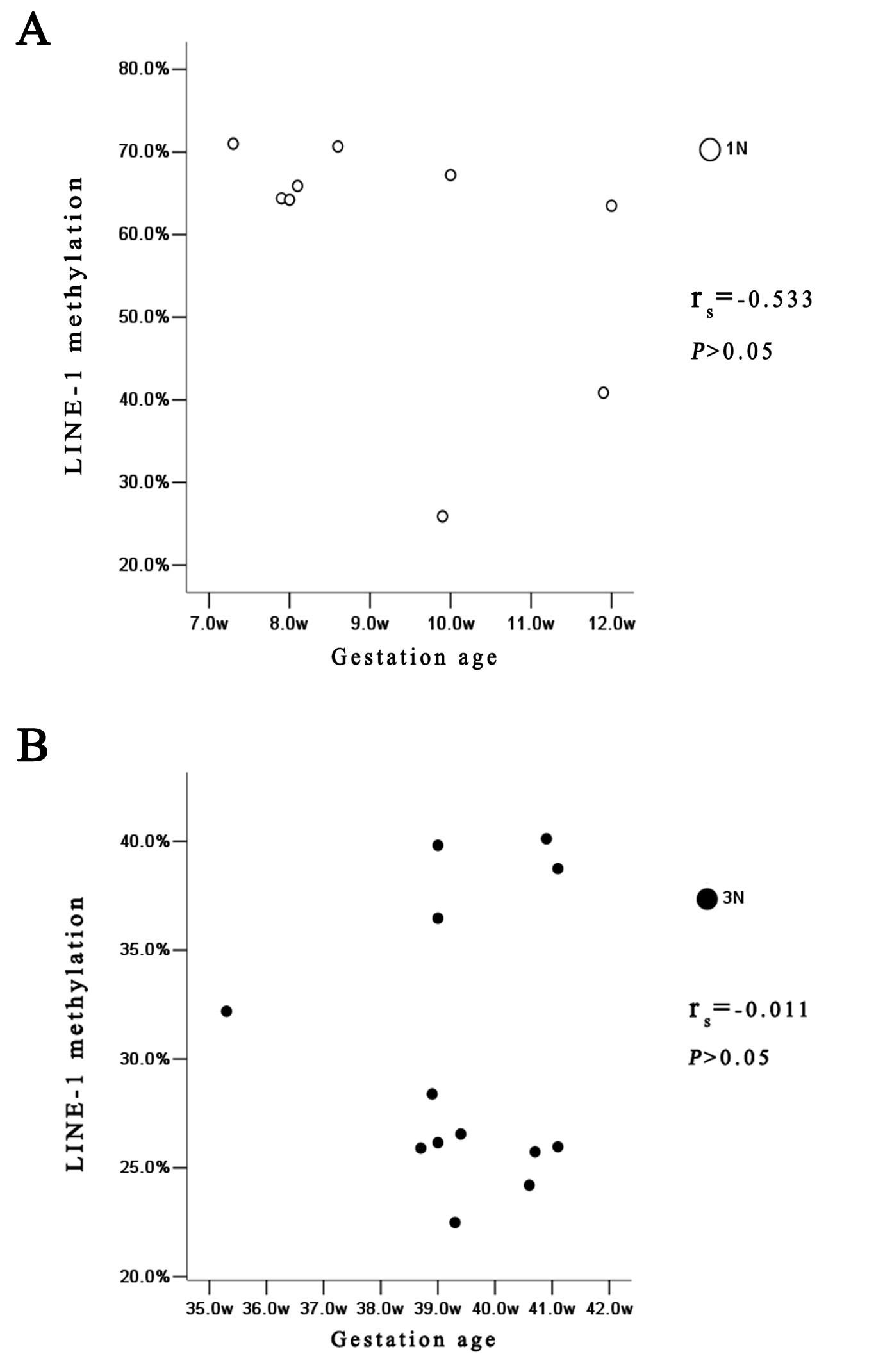

Supplement: Figure S3 — Spearman correlation analyses showed that no significant correlation between LINE-1 methylation levels and gestation ages were detected in either 1N (A) or 3N (B) placentas when the two groups were analyzed separately. (TIF) [file pone.0096994.s003.tif]
